# Future anxiety and psychological wellbeing among sport management students: the indirect role of mental toughness and the joint moderating role of age and family economic status

**DOI:** 10.3389/fpsyg.2026.1955354

**Published:** 2026-10-15

**Authors:** Seda Nur Turhal, Faik Öz, Oğuzhan Gül, Yunus Şahinler, İsmail Belli, Mehmet Bayansalduz

**Affiliations:** 1 Department of Sports Management, Faculty of Sport Sciences, Avrasya University, Trabzon, Türkiye; 2 Red and River Digital Publishing and Trade Limited Company, Rize, Türkiye; 3 Department of Health, Culture and Sports, Sivas Cumhuriyet University, Sivas, Türkiye; 4 Faculty of Sport Sciences, Department of Exercise and Sport Sciences, İstanbul Gelişim University, İstanbul, Türkiye; 5 Department of Exercise and Sport Sciences, Faculty of Sport Sciences, Haliç University, İstanbul, Türkiye; 6 Faculty of Sport Sciences, Department of Sport Management, İstanbul Topkapı University, İstanbul, Türkiye

**Keywords:** age, family economic status, future anxiety, mental toughness, psychological wellbeing, sport management students

## Abstract

**Introduction:**

This study examined the association between future anxiety and psychological wellbeing, the indirect association through mental toughness, and the joint moderating role of age and family economic status in the relationship between future anxiety and mental toughness among sport management students.

**Methods:**

A cross-sectional correlational design was used with 367 students enrolled in sport management departments at four public universities in Türkiye. Data were collected using the Future Anxiety Scale for University Students, the Mental Toughness Inventory, and the Psychological Wellbeing Scale. Pearson correlation, observed-variable path analysis, and three-way interaction analysis were conducted.

**Results:**

Future anxiety was negatively associated with psychological wellbeing (*r* = −0.40, *p* < 0.001) and mental toughness (*r* = −0.35, *p* < 0.001), whereas mental toughness was positively associated with psychological wellbeing (*r* = 0.75, *p* < 0.001). The indirect association between future anxiety and psychological wellbeing through mental toughness was statistically significant (*β* = −0.240, 95% BCa bootstrap CI [−0.336, −0.160]), while the direct association remained significant (*β* = −0.165, *p* < 0.001), indicating that mental toughness accounted for part of the association. The three-way interaction among future anxiety, age, and family economic status was statistically significant in predicting mental toughness (*B* = 0.550, *p* = 0.006). Future anxiety was negatively associated with mental toughness across all age and family economic status groups. The largest observed negative slope was found among students aged 22 years or older whose family income was lower than their family expenses (*B* = −0.620, *p* < 0.001), whereas the smallest observed negative slope was found among students aged 18–21 years in the same economic group (*B* = −0.180, *p* = 0.041).

**Discussion:**

These findings identify mental toughness as an important psychological resource associated with psychological wellbeing and indicate that age and family economic status jointly condition the strength of the relationship between future anxiety and mental toughness. Given the cross-sectional design, the findings should be interpreted as statistical associations rather than evidence of causal relationships.

## 1 Introduction

Future anxiety is an important psychological variable associated with university students’ uncertainty and negative expectations about the future. Studies conducted with university students have shown that future anxiety is negatively associated with psychological wellbeing and is related to various dimensions of wellbeing, including positive emotion, engagement, meaning, and accomplishment (Mukaromah and Sastri, 2025; Öztekin, 2025). Future anxiety is a particularly relevant psychological construct for sport management students because their transition from university to employment is often accompanied by uncertainty regarding professional recognition, limited and competitive employment opportunities, and ambiguity about possible career pathways within the sport industry. Research conducted specifically with sport management students has shown that concerns about employment, professional uncertainty, sectoral expectations, and future career prospects are prominent features of their educational experience. Araç Ilgar and Cihan (2019) found that sport management students expressed considerable uncertainty about post-graduation employment, professional status, and their future within the sport sector. Similarly, Pepe and Aydın (2022) reported high overall levels of professional anxiety among sport management students, including employment-related, economic, social, and professional-development concerns. Comparative evidence further indicates that final-year sport management students may experience greater unemployment anxiety than students enrolled in other sport sciences departments, which may partly reflect perceived limitations in employment opportunities and insufficient recognition of the sport management profession (Yaşar and Turgut, 2020). Psychological resources related to career development may also be important in this population. Career decision-making self-efficacy among sport management students has been associated with adaptive personal competencies such as time-management skills (Onağ et al., 2021), while self-efficacy has also been shown to contribute to career interests, outcome expectations, and career intentions (Chan et al., 2018). Taken together, these findings suggest that sport management students constitute a theoretically and practically relevant population for examining future anxiety and psychological wellbeing. Although future anxiety may encompass concerns related to employment and career prospects, it represents a broader construct involving uncertainty and negative expectations about future life circumstances. Career anxiety and employment anxiety are therefore treated here as related but conceptually distinct constructs, and the present study did not directly measure either of them.

Mental toughness is regarded as an important psychological attribute that may support adaptation and effective functioning when individuals encounter pressure and psychological strain. Evidence from university students indicates that mental toughness is positively associated with psychological wellbeing and is a significant predictor of wellbeing (Stamp et al., 2015). A similar relationship has been reported among university student-athletes, with sport mental toughness being positively associated with psychological wellbeing (Miçooğulları et al., 2017). Beyond these direct associations, mental toughness has also been examined as a psychological resource linking psychosocial factors to wellbeing. Among international students, mental toughness mediated the relationship between adult attachment and psychological wellbeing, while the association between positive childhood experiences and wellbeing was also partly explained by mental toughness (Jin and Wang, 2018; Shaw et al., 2023). In the context of the COVID-19 pandemic and job loss, higher mental toughness was associated with lower levels of depression, anxiety, and stress, supporting its protective role against psychological strain (Mojtahedi et al., 2021). Nevertheless, the indirect role of mental toughness in the association between future anxiety and psychological wellbeing among university students remains insufficiently understood.

Age and family economic status are among the demographic factors that may shape how university students perceive and respond to future-related uncertainty. Previous studies have reported associations between age and future anxiety, although findings concerning the direction and consistency of these associations have varied across samples (Öztekin, 2025; Rabei et al., 2020). Limited economic resources may also increase vulnerability to future-related concerns by restricting students’ capacity to cope with anticipated demands. Evidence indicating that the likelihood of future anxiety increases as household income decreases highlights the importance of economic stability in shaping concerns about the future (Jung et al., 2025). Related research has also shown that economic constraints may weaken work volition and perceptions of obtaining decent work, while more negative perceptions of future employment opportunities are associated with greater employment anxiety (Wan et al., 2024). Qualitative evidence similarly suggests that occupational uncertainty, difficulties in planning for the future, and career-related pressure may shape students’ experiences of career anxiety (Pisarik et al., 2017). Although age and economic resources have generally been examined separately, these factors may operate jointly as contextual conditions that influence how strongly future anxiety is associated with personal psychological resources such as mental toughness. Their joint moderating role in the relationship between future anxiety and mental toughness therefore remains insufficiently understood.

The present study extends the existing literature in three theoretically relevant ways. First, it draws on Conservation of Resources Theory (Hobfoll, 2001; Halbesleben et al., 2014; Hobfoll et al., 2018) to frame the associations among future anxiety, mental toughness, and psychological wellbeing among sport management students by conceptualizing future anxiety as a perceived threat to psychological resources and mental toughness as a personal resource associated with adaptive psychological functioning. Second, rather than examining future anxiety, mental toughness, and psychological wellbeing as isolated correlates, the study integrates these variables within a theoretically specified indirect-association framework in which mental toughness is examined as a statistical intermediary between future anxiety and psychological wellbeing. Third, the study examines whether age and family economic status jointly condition the association between future anxiety and mental toughness. This joint moderation approach considers whether combinations of age-related and economic circumstances are associated with differences in the strength of this relationship. By integrating a future-oriented psychological stressor, a personal psychological resource, and contextual demographic conditions within the same analytical framework, the study offers a more differentiated framework for understanding psychological functioning among sport management students. Accordingly, the present study examines the direct association between future anxiety and psychological wellbeing, the indirect association through mental toughness, and the joint moderating role of age and family economic status in the association between future anxiety and mental toughness.

### 1.1 Theoretical framework

Conservation of Resources Theory proposes that individuals strive to acquire, protect, and maintain valued resources. Stress occurs when these resources are threatened, lost, or fail to increase despite continued investment (Hobfoll, 2001; Halbesleben et al., 2014). Resource loss is considered more psychologically damaging than an equivalent resource gain, making individuals with limited resources more vulnerable to further strain (Hobfoll et al., 2018). Among university students, resource loss has been associated with higher levels of anxiety and psychological distress (Kelada et al., 2023). Accordingly, the theory provides an appropriate basis for conceptualizing future anxiety as a threat to resources and mental toughness as a personal psychological resource.

Future anxiety reflects uncertainty, fear, and negative expectations regarding forthcoming life events. When the future is perceived as threatening, students may experience greater psychological strain and a reduced capacity to cope effectively with uncertainty. Previous evidence has linked higher levels of future anxiety to lower psychological wellbeing and poorer resilience-related functioning in student populations (Öztekin, 2025; Bayram and Pala, 2025; Dalmış et al., 2025). From the perspective of Conservation of Resources Theory, future anxiety can therefore be conceptualized as a perceived threat to psychological resources that may undermine students’ capacity to maintain adaptive functioning.

Mental toughness refers to the capacity to maintain confidence, emotional control, attention, and persistence under pressure (Gucciardi et al., 2015). These characteristics can be viewed as personal psychological resources that support adaptive functioning when individuals encounter stress and uncertainty. Previous studies have shown that mental toughness is positively associated with psychological wellbeing and negatively associated with anxiety and psychological distress (Stamp et al., 2015; Miçooğulları et al., 2017; Lin et al., 2017; Naden et al., 2023). From the perspective of Conservation of Resources Theory, mental toughness may therefore help students preserve their psychological functioning when future-related concerns threaten their available resources.

Mental toughness may help explain the association between future anxiety and psychological wellbeing. From a Conservation of Resources perspective, future-related uncertainty and negative expectations may be conceptualized as perceived threats to valued psychological resources (Hobfoll, 2001; Hobfoll et al., 2018). Mental toughness, which encompasses capacities such as confidence, persistence, emotional regulation, and effective functioning under pressure, can be conceptualized as a personal psychological resource that is relevant when individuals face uncertainty and psychological strain (Gucciardi et al., 2015). Higher mental toughness has been associated with better psychological wellbeing and lower psychological distress, and previous studies have identified mental toughness as an intermediary variable in associations linking psychosocial experiences with wellbeing-related outcomes (Jin and Wang, 2018; Shaw et al., 2023). Related evidence among university students has also reported indirect pathways involving mental toughness and anxiety-related outcomes (Qin et al., 2024). On this theoretical basis, the present study specifies an indirect association in which greater future anxiety is associated with lower mental toughness, which is in turn associated with lower psychological wellbeing. Because the study is cross-sectional, this indirect association is interpreted as a theoretically specified pattern of statistical relationships rather than as evidence of a temporal or causal mechanism.

The association between future anxiety and mental toughness may vary according to students’ age and family economic status. Age may influence how immediate and consequential future-related decisions, graduation, and career transitions are perceived, whereas family economic status may reflect differences in the material resources available to manage uncertainty and anticipated demands. Previous research has reported associations between age and future anxiety, although the direction and strength of these associations have varied across samples (Jannini et al., 2024). Economic conditions at the household level have also been associated with future anxiety and related forms of future-oriented concern (Jung et al., 2025; Koprowicz and Gumowska, 2022). From a Conservation of Resources perspective, these two factors may operate jointly because the psychological significance of future-related uncertainty may depend not only on the perceived immediacy of future transitions but also on the resources available to respond to them. Accordingly, the present study examines whether the strength of the association between future anxiety and mental toughness differs across combinations of age and family economic status.

### 1.2 Hypotheses

*H1*. Future anxiety is negatively associated with psychological wellbeing among sport management students.

*H2*. Future anxiety is negatively associated with mental toughness among sport management students.

*H3*. Mental toughness is positively associated with psychological wellbeing after controlling for future anxiety.

*H4*. Future anxiety is indirectly associated with psychological wellbeing through mental toughness, such that higher future anxiety is associated with lower mental toughness, which in turn is associated with lower psychological wellbeing.

*H5*. The relationship between future anxiety and mental toughness varies according to the joint moderating role of age and family economic status.

## 2 Methods

### 2.1 Research design

This study employed a cross-sectional correlational design to examine the relationships among future anxiety, mental toughness, and psychological wellbeing among sport management students. In the indirect-association model, future anxiety was specified as the predictor, psychological wellbeing as the outcome, and mental toughness as the statistical intermediary. A separate moderation model was used to examine whether the relationship between future anxiety and mental toughness varied according to the joint influence of age and family economic status. Accordingly, path coefficients corresponding to the direct and indirect associations were estimated in the indirect-association analysis, whereas two-way and three-way interaction terms were examined in the moderation analysis. Given the cross-sectional nature of the study, the estimated paths were interpreted as statistical associations rather than as evidence of causal relationships.

### 2.2 Participants

The participants comprised 367 students enrolled in the sport management departments of Çorum Hitit University, Ondokuz Mayıs University, Tokat Gaziosmanpaşa University, and Sivas Cumhuriyet University. All four participating institutions were public universities. Participants were recruited using convenience sampling. The online data collection form was distributed to students through academic staff and student representatives in the relevant departments. Participation was voluntary. The inclusion criteria were being actively enrolled in a sport management department, having sufficient Turkish reading proficiency to understand the items in the data collection form, and completing the form in full. A sensitivity power analysis was conducted for a multiple regression model with seven predictors. Given the final sample size of 367, *α* = 0.05, and a statistical power of 0.80, the study was capable of detecting an overall effect size of approximately Cohen’s *f^2^* = 0.04.

### 2.3 Measures

#### 2.3.1 Psychological wellbeing scale

The Psychological wellbeing Scale, developed by Diener et al. (2010) and adapted into Turkish by Telef (2013), was used to assess participants’ psychological wellbeing. The scale is a unidimensional eight-item instrument designed to assess socio-psychological functioning, including positive relationships, a meaningful and purposeful life, competence, self-esteem, optimism, and engagement in daily activities. Items are rated on a seven-point Likert-type scale ranging from 1 = Strongly disagree to 7 = Strongly agree. The scale contains no reverse-scored items. In the original scoring procedure, item scores are summed to yield a total score ranging from 8 to 56, with higher scores indicating higher levels of psychological wellbeing. In Telef’s (2013) Turkish adaptation, the single-factor structure was supported, and the Cronbach’s alpha internal consistency coefficient was reported as 0.80. In the present study, the scale score was calculated as the mean of the eight items, and the Cronbach’s alpha coefficient was 0.89.

#### 2.3.2 Mental toughness inventory

The Mental Toughness Inventory, developed by Gucciardi et al. (2015) and adapted into Turkish by Etlioğlu and Yılmaz (2025), was used to assess participants’ mental toughness. The inventory is a unidimensional eight-item measure designed to assess confidence in achieving goals, regulation of attention and emotions, persistence, coping with challenges, and the ability to maintain a positive outlook. Items are rated on a seven-point Likert-type scale. The inventory contains no reverse-scored items, and higher scores indicate greater mental toughness. In the Turkish adaptation conducted by Etlioğlu and Yılmaz (2025), the single-factor structure was supported, and the Cronbach’s alpha internal consistency coefficient was reported as 0.926. In the present study, the inventory score was calculated as the mean of the eight items, and the Cronbach’s alpha coefficient was 0.92.

#### 2.3.3 Future anxiety scale for university students

The Future Anxiety Scale for University Students, developed by Geylani and Çiriş Yıldız (2022), was used to assess participants’ future anxiety. The scale consists of 19 items grouped into two subdimensions: Fear of the Future and Hopelessness about the Future. Items are rated on a five-point Likert-type scale ranging from 1 = Never to 5 = Always. Items 2, 4, 6, 8, 10, and 12 are reverse-scored. After reverse coding, higher scores indicate greater levels of future anxiety. In the scale development study, Cronbach’s alpha internal consistency coefficients were reported as 0.91 for the total scale, 0.95 for the Fear of the Future subdimension, and 0.88 for the Hopelessness about the Future subdimension. In the present study, the overall future anxiety score was calculated as the mean of the 19 items after reverse coding, and the Cronbach’s alpha coefficient was 0.92.

### 2.4 Data collection procedure

Data were collected across the fall and spring semesters of the 2025–2026 academic year using an online data collection form administered to students enrolled in sport management departments at four public universities in Türkiye. The survey link was distributed through departmental student communication groups, institutional email lists, and student representatives. At the beginning of the form, participants were informed about the purpose of the study, the voluntary nature of participation, the anonymity of their responses, and their right to withdraw at any time without providing a reason. No personally identifiable information was collected. To reduce social desirability bias, participants were informed that there were no right or wrong answers and were encouraged to respond honestly. Participants who provided informed consent completed the Personal Information Form, the Psychological wellbeing Scale, the Mental Toughness Inventory, and the Future Anxiety Scale for University Students. Incomplete forms were excluded from the analyses, resulting in a final sample of 367 participants.

### 2.5 Ethical considerations

Ethical approval for this study was obtained from the Sivas Cumhuriyet University Educational Sciences Research Proposal Ethics Evaluation Board. The research proposal was reviewed and approved as being in accordance with the principles of scientific research ethics (Application No. 24_04_17; Document Date: April 29, 2024; Document No. E-50704946-100-420912). All participants were informed about the purpose of the study, and informed consent was obtained prior to participation.

### 2.6 Data analysis

Data were analyzed using R statistical software (R Core Team, 2024). Before conducting the analyses, the dataset was examined for missing values, outliers, and distributional characteristics. Participants’ demographic characteristics were summarized using frequencies and percentages, whereas continuous variables were described using means and standard deviations. The distributions of the scale scores were evaluated using skewness and kurtosis coefficients, and the internal consistency of the measures was assessed using Cronbach’s alpha coefficients. Bivariate associations among future anxiety, mental toughness, and psychological wellbeing were examined using Pearson correlation analysis. Prior to the regression-based analyses, diagnostic checks were conducted to evaluate linearity, homoscedasticity, residual normality, multicollinearity, and influential observations. Visual inspection of residual-versus-fitted plots did not indicate substantial departures from linearity or homoscedasticity, and the Q–Q plots showed no serious deviations from residual normality. Standardized residuals ranged approximately between −2.71 and 2.84, with no observations exceeding the conventional ±3.00 threshold. Variance inflation factor values ranged from 1.08 to 2.16, indicating no problematic multicollinearity. The maximum Cook’s distance was 0.083, and no case exerted undue influence on the estimated regression coefficients. The diagnostic checks indicated that the assumptions underlying the regression and interaction analyses were adequately satisfied.

The indirect association through mental toughness was examined using observed-variable path analysis with the lavaan package. Future anxiety was specified as the predictor, psychological wellbeing as the outcome, and mental toughness as the statistical intermediary. Path coefficients corresponding to the direct, indirect, and total associations were estimated. The statistical significance of the indirect association was assessed using a 95% bias-corrected and accelerated bootstrap confidence interval based on 5,000 bootstrap samples. The indirect association was considered statistically significant when the bootstrap confidence interval did not include zero.

A separate moderation analysis was conducted in R to determine whether the association between future anxiety and mental toughness varied according to the joint influence of age and family economic status. Future anxiety was mean-centered before the interaction terms were computed, and the conditional effects were examined using the interactions package (Long, 2022). Age was coded into two groups: 18–21 years and 22 years or older. For the moderation analysis, this regrouping was used to avoid very small age-group cells, particularly because the original 25–27-year and 28-years-or-older groups included relatively few participants (*n* = 15 and *n* = 32, respectively). Family economic status was initially assessed using three categories: family income lower than expenses, equal to expenses, and higher than expenses. For the moderation analysis, the categories of income equal to expenses and income higher than expenses were combined to distinguish participants reporting a relative financial shortfall from those whose reported family income was sufficient to meet or exceed family expenses. Accordingly, family economic status was recoded as income lower than expenses and income equal to or higher than expenses. These regrouping decisions were used to obtain sufficiently populated and interpretable groups for the moderation analysis; however, the potential loss of information resulting from categorical recoding is acknowledged as a limitation. The model included the main effects of future anxiety, age, and family economic status; all two-way interaction terms; and the three-way interaction term among future anxiety, age, and family economic status. Multicollinearity was assessed using variance inflation factor values. When the three-way interaction was statistically significant, conditional simple slopes were estimated for the four combinations of age and family economic status. Statistical significance was set at *p* < 0.05.

## 3 Results

A total of 367 sport management students participated in the study. Of the participants, 219 were female (59.7%) and 148 were male (40.3%). More than half of the participants were aged 18–21 years (52.6%), followed by those aged 22–24 years (34.6%), 28 years or older (8.7%), and 25–27 years (4.1%). Regarding family economic status, 141 participants (38.4%) reported that their family income was equal to their family expenses, 114 participants (31.1%) reported that their family income was greater than their family expenses, and 112 participants (30.5%) reported that their family income was less than their family expenses. For the moderation analyses, age was regrouped into two categories, 18–21 years and 22 years or older, whereas family economic status was regrouped into income less than family expenses and income equal to or greater than family expenses (Table 1).

**Table 1:** Demographic characteristics of the participants.

Variable	Category	*n*	%
Gender	Male	148	40.3
	Female	219	59.7
Age	18–21	193	52.6
	22–24	127	34.6
	25–27	15	4.1
	28 years and older	32	8.7
Family economic status	Income less than expenses	112	30.5
	Income equal to expenses	141	38.4
	Income greater than expenses	114	31.1

For the moderation analyses, age was regrouped as 18–21 years and 22 years or older, whereas family economic status was regrouped as family income less than family expenses and family income equal to or greater than family expenses.

As shown in Table 2, the mean scores for psychological wellbeing and mental toughness were 5.49 (SD = 1.03) and 5.50 (SD = 1.07), respectively, whereas the mean future anxiety score was 2.65 (SD = 0.75). The internal consistency coefficients ranged from 0.89 to 0.92, indicating high reliability across the three measures. Psychological wellbeing was strongly and positively correlated with mental toughness (*r* = 0.75, *p* < 0.001). Future anxiety was negatively correlated with psychological wellbeing (*r* = −0.40, *p* < 0.001) and mental toughness (*r* = −0.35, *p* < 0.001). These results indicate that higher future anxiety was associated with lower levels of mental toughness and psychological wellbeing.

**Table 2:** Descriptive statistics, reliability coefficients, and correlations among the study variables.

Variable	*M*	SD	Skewness	Kurtosis	*α*	1	2	3
1. Psychological wellbeing	5.49	1.03	−1.61	3.37	0.89	–		
2. Mental toughness	5.50	1.07	−1.51	3.43	0.92	0.75^***^	–	
3. Future anxiety	2.65	0.75	−0.04	−0.21	0.92	−0.40^***^	−0.35^***^	–

*N* = 367. *M* = mean; SD = standard deviation; *α* = Cronbach’s alpha coefficient. ^***^p < 0.001.

As shown in Table 3, future anxiety was negatively associated with mental toughness (*β* = −0.347, *p* < 0.001), whereas mental toughness was positively associated with psychological wellbeing after controlling for future anxiety (*β* = 0.692, *p* < 0.001). The indirect association between future anxiety and psychological wellbeing through mental toughness was statistically significant (*β* = −0.240, 95% BCa bootstrap CI [−0.336, −0.160]). The direct association between future anxiety and psychological wellbeing remained statistically significant after mental toughness was included in the model (*β* = −0.165, *p* < 0.001). Thus, mental toughness accounted for part of the association between future anxiety and psychological wellbeing. The total association between future anxiety and psychological wellbeing was also statistically significant (*β* = −0.405, *p* < 0.001) (Figure 1).

**Table 3:** Indirect association analysis results.

Association/path	*β*	SE	95% BCa bootstrap CI	*p*
Future anxiety → Mental toughness (a path)	−0.347	0.059	[−0.461, −0.233]	<0.001
Mental toughness → Psychological wellbeing (b path)	0.692	0.059	[0.573, 0.805]	<0.001
Future anxiety → Psychological wellbeing, direct association (c′ path)	−0.165	0.038	[−0.240, −0.090]	<0.001
Indirect association through mental toughness (a × b)	−0.240	0.045	[−0.336, −0.160]	<0.001
Total association (c path)	−0.405	0.052	[−0.512, −0.308]	<0.001

**Figure 1:**
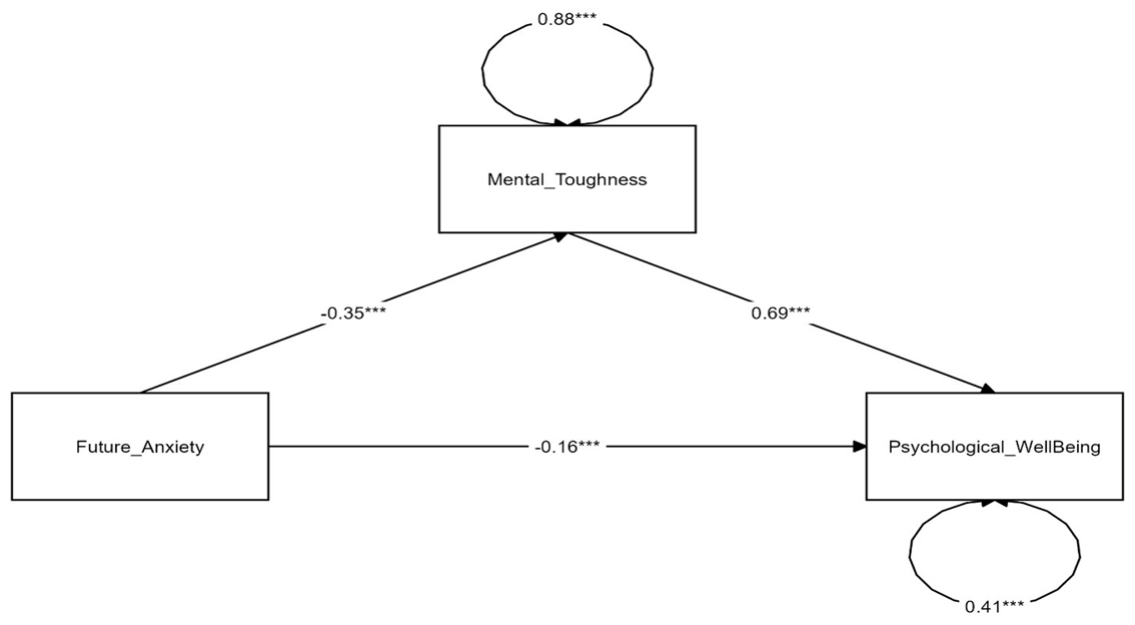
Indirect association model linking future anxiety, mental toughness, and psychological wellbeing. Standardized path coefficients are presented. Values shown above the endogenous variables represent residual variances. The indirect association between future anxiety and psychological wellbeing through mental toughness was statistically significant (*β* = −0.240, 95% BCa bootstrap CI [−0.336, −0.160]). ^***^p < 0.001.

The three-way interaction among future anxiety, family economic status, and age was statistically significant in predicting mental toughness (*B* = 0.550, *p* = 0.006). Conditional simple slope analyses indicated that future anxiety was negatively associated with mental toughness across all combinations of age and family economic status. The smallest observed negative slope was found among students aged 18–21 years whose family income was less than their family expenses (*B* = −0.180, *p* = 0.041), whereas the largest observed negative slope was found among students aged 22 years or older whose family income was less than their family expenses (*B* = −0.620, *p* < 0.001). Future anxiety was also negatively associated with mental toughness among students aged 18–21 years whose family income was equal to or greater than their family expenses (*B* = −0.350, *p* < 0.001) and among students aged 22 years or older whose family income was equal to or greater than their family expenses (*B* = −0.240, *p* = 0.009). Taken together, the conditional effects show that the negative association between future anxiety and mental toughness was present in all four age-by-economic-status combinations, while the pattern of the slopes varied across these combinations. Specifically, the age-related difference in the association was substantially more pronounced among students whose family income was below expenses than among those whose family income was equal to or greater than expenses, consistent with the significant three-way interaction. These results indicate that the strength of the negative association between future anxiety and mental toughness varied according to the joint moderating role of age and family economic status (Table 4; Figure 2).

**Table 4:** Three-way interaction effects and conditional simple slopes.

Model component/group	*B*	SE	95% CI	*p*
Main and interaction effects				
Future anxiety	−0.180	0.088	[−0.353, −0.007]	0.041
Family economic status	0.184	0.079	[0.029, 0.339]	0.021
Age	0.126	0.073	[−0.017, 0.269]	0.084
Future anxiety × Family economic status	−0.170	0.079	[−0.325, −0.015]	0.032
Future anxiety × Age	−0.440	0.102	[−0.640, −0.240]	<0.001
Family economic status × Age	−0.156	0.094	[−0.340, 0.028]	0.096
Future anxiety × Family economic status × Age	0.550	0.199	[0.160, 0.940]	0.006
Conditional simple slopes of future anxiety predicting mental toughness				
Age 18–21, income less than expenses	−0.180	0.088	[−0.353, −0.007]	0.041
Age 18–21, income equal to or greater than expenses	−0.350	0.075	[−0.497, −0.203]	<0.001
Age 22 or older, income less than expenses	−0.620	0.085	[−0.787, −0.453]	<0.001
Age 22 or older, income equal to or greater than expenses	−0.240	0.092	[−0.420, −0.060]	0.009

*N* = 367. Unstandardized regression coefficients are reported. Mental toughness was the dependent variable. Future anxiety was mean-centered before the interaction terms were calculated. Age was coded as 0 = 18–21 years and 1 = 22 years or older, whereas family economic status was coded as 0 = income less than expenses and 1 = income equal to or greater than expenses. The overall regression model was statistically significant, *R^2^* = 0.19, *F* (7, 359) = 12.14, *p* < 0.001.

**Figure 2:**
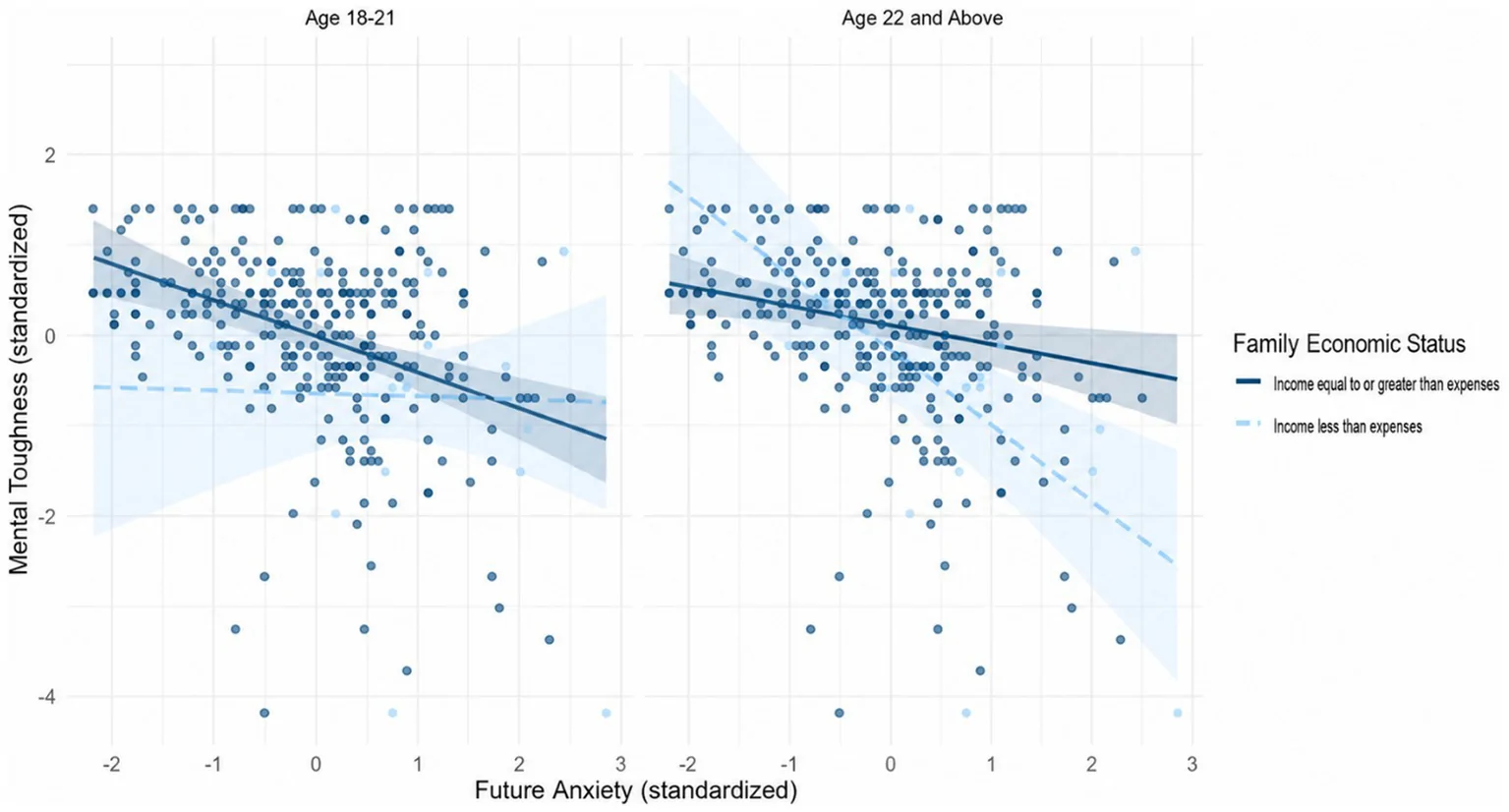
Joint moderating effect of age and family economic status on the relationship between future anxiety and mental toughness. Future anxiety was mean-centered before the interaction terms were calculated. The three-way interaction among future anxiety, family economic status, and age was statistically significant in predicting mental toughness (*B* = 0.550, *p* = 0.006). Among students aged 18–21 years, future anxiety was negatively associated with mental toughness both when family income was less than family expenses (*B* = −0.180, *p* = 0.041) and when family income was equal to or greater than family expenses (*B* = −0.350, *p* < 0.001). Among students aged 22 years or older, future anxiety was negatively associated with mental toughness both when family income was less than family expenses (*B* = −0.620, *p* < 0.001) and when family income was equal to or greater than family expenses (*B* = −0.240, *p* = 0.009). Shaded areas represent 95% confidence intervals.

## 4 Discussion

Future anxiety was negatively associated with psychological wellbeing and mental toughness among sport management students, supporting H1 and H2. The first finding is consistent with previous research linking future anxiety to lower psychological or subjective wellbeing, greater psychological strain, and poorer quality of life (Bouclaous et al., 2023; Jannini et al., 2024; Satici et al., 2025; Sun, 2026). Some evidence suggests that this relationship may vary according to how anxiety and wellbeing are conceptualized. Akkoç et al. (2025) found that psychological wellbeing mediated the relationship between inhibitory anxiety and academic life satisfaction, whereas the same mediating pattern was not observed for prospective anxiety. This difference may reflect the narrower scope of prospective anxiety and the different roles assigned to psychological wellbeing across models. The second finding is consistent with previous research linking higher mental toughness with lower anxiety and more favourable psychological functioning (Demir et al., 2025; Mojtahedi et al., 2021; Naden et al., 2023). Related studies also indicate that future anxiety and employment anxiety are associated with weaker resilience-related psychological resources among university students and early-career populations (Dalmış et al., 2025; Satici et al., 2025; Wang and Zhang, 2025; Yang et al., 2025). Although mental toughness and psychological resilience are distinct constructs, both involve capacities that may support adaptive coping with stress and uncertainty. Among sport management students, uncertainty regarding post-graduation employment, professional roles, and economic security may provide relevant contextual background for these associations; however, because these factors and employment anxiety were not directly measured, these interpretations should remain tentative. Mental toughness was positively associated with psychological wellbeing among sport management students, supporting H3. This finding is consistent with previous research linking higher mental toughness with greater psychological wellbeing, better adjustment, and lower psychological distress across student and athlete samples (Akoğlu et al., 2024; Jin and Wang, 2018; Lin et al., 2017; Miçooğulları et al., 2017; Naden et al., 2023; Stamp et al., 2015). Among sport management students, characteristics associated with mental toughness, such as confidence, persistence, and effective regulation under pressure, may be relevant to psychological wellbeing; however, these specific processes were not directly measured in the present study.

The observed indirect association was consistent with H4, indicating that mental toughness accounted for part of the association between future anxiety and psychological wellbeing. This pattern is consistent with previous evidence indicating that psychological resources and related intermediary processes may play a role in associations involving perceived stress, anxiety, psychosocial experiences, and wellbeing-related outcomes (Jin and Wang, 2018; Qin et al., 2024; Shaw et al., 2023; Toktaş et al., 2026; Yılmaz et al., 2026). Related models also suggest that resilience-related resources may attenuate the adverse association between future anxiety and psychological wellbeing (Bayram and Pala, 2025; Satici et al., 2025). However, mental toughness has not emerged as a significant intermediary variable in every context, suggesting that its explanatory role may depend on the stressor and outcome examined (Ozcan, 2022). Among sport management students, higher mental toughness may therefore represent a psychological resource associated with better psychological wellbeing when future-related concerns are high. Because the data were cross-sectional, this indirect association should be interpreted as a statistical pattern rather than evidence of a causal or temporal process.

The relationship between future anxiety and mental toughness varied according to the joint moderating role of age and family economic status, consistent with H5. The conditional effects indicated that the negative association between future anxiety and mental toughness differed across age and family economic status groups. The largest observed negative slope was found among students aged 22 years or older whose family income was less than their family expenses. Previous evidence indicates that socioeconomic disadvantage and lower perceived social status are associated with greater future anxiety, whereas findings concerning the role of age are not fully consistent across populations (Jung et al., 2025; Lorini et al., 2023; Rabei et al., 2020; Saraç and Yıldız, 2025). Employment-related uncertainty and perceived limitations in future work opportunities may also provide relevant contextual background for future-oriented anxiety among university students (Wan et al., 2024; Yorulmaz, 2019). The comparatively larger negative slope observed among students aged 22 years or older whose family income was less than their family expenses may reflect the combined relevance of more immediate career-transition demands and limited economic resources. However, these factors were not measured directly, and this interpretation should therefore be regarded as tentative.

Taken together, the findings indicate that future anxiety is associated with poorer psychological wellbeing among sport management students, partly through its negative association with mental toughness. The strength of the association between future anxiety and mental toughness also differed across age and family economic status groups. Mental toughness therefore appears to be an important psychological resource whose role may vary according to students’ socioeconomic and age-related characteristics. These findings should nevertheless be interpreted cautiously given the cross-sectional design and the categorical coding of age and family economic status.

## 5 Practical implications

The findings suggest that support services for sport management students may consider addressing future-related uncertainty and psychological resources related to mental toughness. Sport management departments could periodically assess students’ future-related concerns and psychological wellbeing while also considering whether career and economic uncertainties contribute to these concerns. Career services may consider structured activities related to realistic career planning, decision-making under uncertainty, confidence under pressure, and the maintenance of professional goals. Although recent experimental evidence indicates that psychological skills training can enhance mental toughness in sport populations (Tutal et al., 2026), the effectiveness of such interventions was not evaluated in the present study. These suggestions are informed by the observed pattern of associations, including the indirect association through mental toughness, but they should not be interpreted as evidence that such interventions would causally improve psychological wellbeing. The findings also suggest that age and family economic status may be relevant contextual factors to consider when planning student support. Students aged 22 years or older whose family income was less than their family expenses showed the largest observed negative slope between future anxiety and mental toughness in the present sample. This subgroup may therefore warrant closer attention in future support planning, although the present findings do not establish which specific services or interventions would be most effective. Sport management departments, university career centres, and psychological counselling units may therefore consider whether support should account for differences in students’ age, economic circumstances, and future-related concerns rather than assuming that identical forms of support are appropriate for all students.

## 6 Limitations and future research

Several limitations should be considered when interpreting the findings. First, the cross-sectional design does not establish the temporal ordering of future anxiety, mental toughness, and psychological wellbeing. The significant indirect association therefore represents a statistical association rather than evidence that future anxiety influences psychological wellbeing through changes in mental toughness over time. Longitudinal studies should examine whether future anxiety predicts subsequent changes in mental toughness and psychological wellbeing among sport management students. Second, future anxiety, mental toughness, and psychological wellbeing were assessed using self-report measures administered at the same time point. This design may have increased shared method variance and response-related covariance among the study variables. Particular caution is warranted regarding the relatively strong association between mental toughness and psychological wellbeing (*r* = 0.75). Although the two constructs are theoretically distinct, this magnitude of association may reflect not only a substantive relationship but also some degree of conceptual overlap and shared measurement characteristics. Consequently, the strength of the estimated association between mental toughness and psychological wellbeing, and therefore the magnitude of the indirect association involving mental toughness, may have been partly influenced by shared construct or method variance. The present study did not include a marker variable, a latent common-method factor, or a dedicated measurement model designed to establish discriminant validity between these constructs; therefore, the relative contribution of substantive covariance and shared method variance cannot be determined from the present data. Future studies should examine the distinctiveness of mental toughness and psychological wellbeing more directly using latent-variable measurement models, comparisons of alternative factor structures, and appropriate indices of discriminant validity, while also incorporating multiple methods, informants, or measurement occasions where feasible. Because the present study assessed general future anxiety rather than career-specific or employment-specific anxiety, the findings should not be interpreted as directly representing students’ career or employment anxiety. Future studies could also use repeated measurements and include indicators of career-related uncertainty, such as perceived employability, employment expectations, and career stress.

## 7 Conclusion

Future anxiety was associated with lower psychological wellbeing among sport management students, with a significant indirect association through mental toughness. The association between future anxiety and mental toughness also differed according to the joint role of age and family economic status, with the largest observed negative slope found among students aged 22 years or older whose family income was less than their family expenses. These findings highlight mental toughness as an important psychological resource for understanding how future-related concerns are associated with students’ psychological functioning. They also suggest that age and family economic status may need to be considered together rather than independently when interpreting this association. The results may inform more targeted support for sport management students, although the cross-sectional design does not permit causal conclusions. Longitudinal studies are needed to examine whether these associations remain stable over time.

## Data Availability

The raw data supporting the conclusions of this article will be made available by the authors, without undue reservation.
